# Fibroblasts in urothelial bladder cancer define stroma phenotypes that are associated with clinical outcome

**DOI:** 10.1038/s41598-019-55013-0

**Published:** 2020-01-14

**Authors:** Artur Mezheyeuski, Ulrika Segersten, Lina Wik Leiss, Per-Uno Malmström, Jiri Hatina, Arne Östman, Carina Strell

**Affiliations:** 1 Department of Immunology, Genetics and Pathology, Uppsala University, Uppsala, Sweden; 2 Department of Surgical Sciences, Uppsala University, Uppsala, Sweden; 3 Department of Oncology-Pathology, Karolinska Institutet, Stockholm, Sweden; 4 Charles University, Faculty of Medicine in Pilsen, Institut of Biology, Plzen, Czech Republic

**Keywords:** Cancer microenvironment, Bladder cancer

## Abstract

Little attention was given to the interaction between tumor and stromal cells in urothelial bladder carcinoma (UBC). While recent studies point towards the existence of different fibroblast subsets, no comprehensive analyses linking different fibroblast markers to UBC patient survival have been performed so far. Through immunohistochemical analysis of five selected fibroblast markers, namely alpha smooth muscle actin (ASMA), CD90/Thy-1, fibroblast activation protein (FAP), platelet derived growth factor receptor-alpha and -beta (PDGFRa,-b), this study investigates their association with survival and histopathological characteristics in a cohort of 344 UBC patients, involving both, muscle-invasive and non-muscle-invasive cases. The data indicates that combinations of stromal markers are more suited to identify prognostic patient subgroups than single marker analysis. Refined stroma-marker-based patient stratification was achieved through cluster analysis and identified a FAP-dominant patient cluster as independent marker for shorter 5-year-survival (HR(95% CI)2.25(1.08–4.67), p = 0.030). Analyses of interactions between fibroblast and CD8a-status identified a potential minority of cases with CD90-defined stroma and high CD8a infiltration showing a good prognosis of more than 80% 5-year-survival. Presented analyses point towards the existence of different stroma-cell subgroups with distinct tumor-modulatory properties and motivate further studies aiming to better understand the molecular tumor–stroma crosstalk in UBC.

## Introduction

The estimated incidence of urinary bladder cancer (UBC) worldwide was about 550.000 new cases and 200.000 deaths in 2018^1^, with highest incidences indicated in developed areas^2^. Given that the prevalence of UBC is several times higher than the incidence, it represents a major burden for health care systems. Carcinomas originating from the urothelium, the inner surface of the bladder, represent the most common type of bladder cancer^3,4^. Approximately 70% of all newly diagnosed cases are classified as non-muscle-invasive bladder cancer disease, while about 30% of patients have already muscle-invasive tumors with 10–15% of them being metastatic^5–8^. Reported 5-year recurrence rates of non-muscle invasive cases range between 40–70% among the highest recurrence rates of solid tumors^3,9^.

Multidisciplinary approaches have been established in order to improve patient management and to decrease the mortality rate, but still treatment strategies for UBC remain controversial. A better understanding of the mechanisms underlying tumor progression is crucial and novel prognostic and predictive markers as well as therapeutic targets need to be identified^10,11^.

Recent research efforts on UBC aimed mostly to decipher molecular drivers of an invasive tumor phenotype through description of genetic varieties, and with the introduction of checkpoint inhibitory immunotherapy, awareness has also risen for tumor infiltrating leukocytes^12,13^. However, little attention was given the interaction of tumor cells and non-leukocytic stromal cells. Cancer-associated fibroblasts (CAFs) are a highly abundant cell-type in most solid tumors. In UBC the abundance of alpha smooth muscle actin (ASMA) and vimentin positive CAFs together with a desmoplastic reaction was described to be most prominent in invasive tumors^14^. Few experimental studies have demonstrated functional evidence for a modulatory role of fibroblasts on urothelial tumor cells *in vitro* and *in vivo*. While the majority of these studies describe pro-tumoral effects of fibroblasts derived growth-factors, like the induction of epithelial to mesenchymal transition and invasion^5,10,15,16^, one study also demonstrated that sonic hedgehog (Shh) activated fibroblasts exhibit rather tumor restraining functions during the transition of *in situ* to invasive UBC^17^. These findings, taken together with studies on fibroblast - tumor cell interactions in other solid tumors, point towards the existence of different fibroblast subsets with different functional roles at various stages of tumor progression and invasion (reviewed in^18–23^). The markers used for the identification of CAFs are highly diverse, probably reflecting the many different potential sources of their origin^18^.

Several fibroblast defining markers including alpha smooth muscle actin (ASMA), CD90/Thy-1, fibroblast activation protein (FAP), platelet derived growth factor receptor-alpha and -beta (PDGFRa, -b) have been studied individually as prognostic markers as well as active functional regulators of tumor progression. ASMA positive fibroblasts -generally considered as myofibroblasts^24^- were found to exhibit tumor restraining properties in a pancreatic tumor model in an immune-regulatory manner^25^, but were also associated with a bad prognosis in certain tumor types^26,27^. FAP positive fibroblasts were linked to immunosuppressive functions within the tumor microenvironment^28–32^. Stromal PDGFRa was found to decrease with tumor progression^33–35^, while a high expression of stromal PDGFRb was associated with a worse prognosis in different solid tumors^36–42^ and drug resistance^43^. Likewise, CD90 defined fibroblasts were adversely associated with clinical outcome^44,45^ but were also discussed as immune-regulatory fibroblasts^46–48^.

Given that cancer-associated fibroblasts represent such a heterogeneous cell population, more extended investigations for associations between the presence of marker-defined fibroblast sub-populations and patient survival or histopathological characteristics for UBC are highly motivated. Above mentioned findings on immune-modulatory properties of fibroblasts support the efforts to identify such specific subpopulations. Especially interactions of fibroblasts with CD8-positive T cells are of high interest in light of recent efforts to advance and refine immunotherapy treatment in UBC. In longer perspective a more detailed understanding of the role of different fibroblast subpopulations in UBC could serve as basis for the description of novel diagnostic and prognostic biomarker or even the identification of novel therapeutic targets or strategies^15^.

The presented study investigates through immunohistochemical staining, the association of ASMA, CD90/Thy-1, FAP, PDGFRa and -b with survival, histopathological characteristics and presence of CD8a positive lymphocytes in a well-annotated cohort of 344 UBC patients involving non-muscle as well as muscle-invasive cases. The data indicates that stroma marker combinations are more suited to identify prognostically distinct patient subgroups than single marker analysis. The study thereby provides correlative support for the existence of different fibroblast subsets with distinct tumor modulatory properties in UBC.

## Results

### Staining evaluation

The analyzed tissue microarray (TMA) included two cores from 357 prospectively collected primary tumors of UBC patients. Follow-up and clinical data was available for 344 patients, with median follow-up time of 44.5 months.

**Figure 1:**
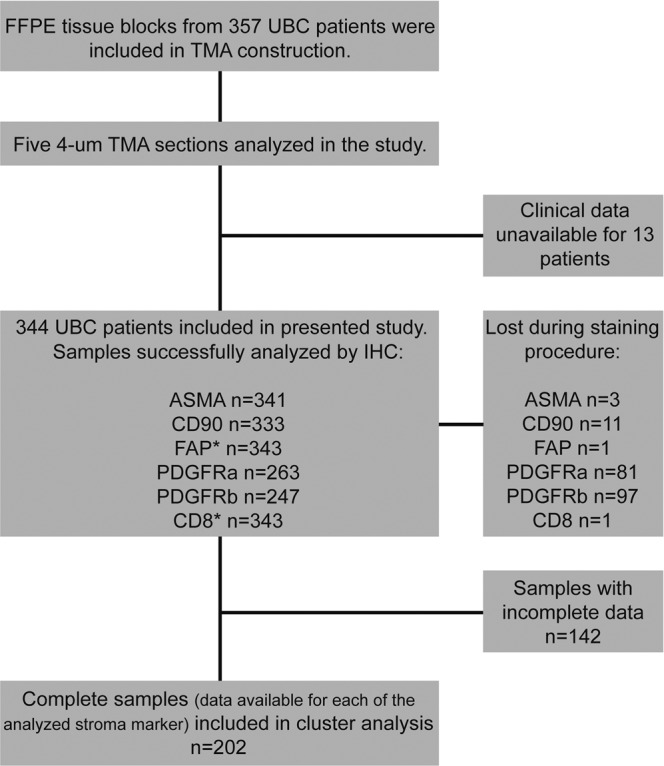
REMARK diagram of sample inclusion. UBC = urinary bladder cancer; TMA = tissue microarray; IHC = immunohistochemistry * FAP and CD8 IHC was performed on the same section.

Five TMA sets were subjected to IHC for ASMA, CD90, FAP with CD8a, PDGFRa and PDGFRb. Of the 344 UBC patient samples, 341 samples were successfully analyzed for ASMA (corresponding to a loss of 0.2% during staining procedure), 333 samples for CD90 (loss of 3.2%), 343 samples for FAP and CD8a (loss of 0.3%), 263 samples for PDGFRa (loss of 23.5%) and 247 samples for PDGFRb (loss of 28.2%) (REMARK diagram Fig. 1).

All investigated markers were evaluated by two independent raters (see Materials and Methods for more detailed information). Stroma markers were scored for intensity as well as positive stroma fraction, using a scale from zero to three and both metrics were multiplied to obtain the overall score per rater (Supplementary Figs. 1 and 2). The frequency of CD8a positive cells was assessed on a scale from zero to four within the tumor and stroma compartment separately and the two metrics were added to obtain the overall score (Supplementary Fig. 2).

The inter-rater agreement^49^ was moderate for CD90 (Cohen’s *kappa* = 0.506) and PDGFRb (*kappa* = 0.599), substantial for ASMA (*kappa* = 0.663) and PDGFRa (*kappa* = 0.620) and excellent for FAP (*kappa* = 0.890) and CD8a (*kappa* = 0.809) (Supplementary Figs. 1 and 2).

### Prognostic value of single stroma markers and association with histopathological tumor characteristics

**Figure 2:**
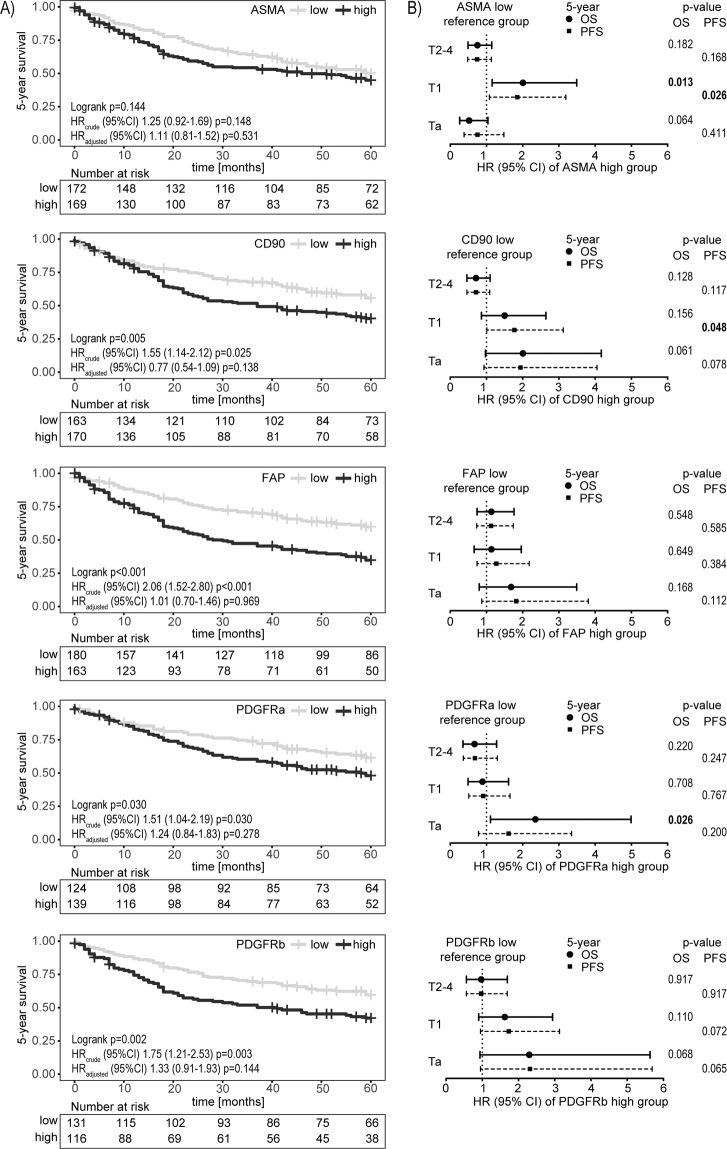
(**A**) Kaplan–Meier analysis of the relationship between “low” (grey line) and “high” (black line) expression of indicated stroma marker using a median cut-off and the 5-year overall survival (OS) of bladder cancer patients. P-values from logrank test evaluate differences between the population survival curves. Crude hazard ratios (HR) are based on Cox proportional hazards regression models and presented with 95% confidence interval (CI) and Wald test. Adjusted HR included tumor stage, grade, age group and gender in the regression model. Tables indicate the number of individuals at risk at the given time points. (**B**) Forest plots of HR for 5-year overall survival (OS; black dot) and progression free survival (PFS; black square) with 95% CI within tumor stage-specific patient subgroups. P-values are based on Wald test. Median cut-off was applied within each stage group to dichotomize patients as “low” or “high” for corresponding marker expression.

For an initial analysis of the relationship between stroma marker expression and the 5-year survival of UBC patients, the analyzed stroma markers were dichotomized into “low” and “high” groups using median cut-off (Fig. 2A). Kaplan-Meier and univariable Cox regression analysis indicated an association between shorter survival time and high expression for the majority of the tested stroma markers namely CD90 (logrank p = 0.005; HR(95%CI) 1.55(1.14–2.12) Wald test p = 0.025), FAP (p < 0.001; HR 2.06(1.52–2.80) p < 0.001), PDGFRa (p = 0.030; HR 1.51(1.04–2.19) p = 0.030) and PDGFRb (p = 0.002; HR 1.75(1.21–2.53) p = 0.003), whereby FAP showed the strongest association (Fig. 2A “HR_crude_”). ASMA expression level did not show an impact on survival (p = 0.144; HR 1.25(0.92–1.69) p = 0.148). After applying Bonferroni correction for multiple testing (critical p-value for five performed tests p < 0.010) only the prognostic associations of FAP and PDGFRb were significant.

However, multivariable analysis adjusting for age group, gender, tumor stage and grade in the Cox regression model did not confirm a significant independent prognostic potential for any of the tested stroma markers (Fig. 2A “HR_adjusted_” and Supplementary Table 1). Age group and stage remained the only strong significant independent prognostic variables.

Subsequent correlation analysis of the single stromal markers with patient- and histopathological tumor characteristics revealed a strong positive correlation between the stroma marker expression level and tumor stage for four of the five stroma markers (CD90, FAP p < 0.001; ASMA, PDGFRb p = 0.001 linear-by-linear association χ^2^-test) but not for PDGFRa (p = 0.141) (Supplementary Table 2; additional confirmation of this finding based on the raw scoring data is shown in Supplementary Fig. 3). High expression of CD90, FAP and PDGFRb was furthermore correlated with higher grade (CD90, FAP p < 0.001, PDGFRb p = 0.001 Fisher’s exact test). A weak association was also observed between age group and CD90 expression (p = 0.028 Fisher’s exact test).

Taking the strong correlation between stroma marker expression and tumor stage into consideration, additional separate survival analyses were performed for the non-muscle invasive stages Ta and T1, as well as for the muscle-invasive stages T2–4. The results for 5-year overall survival indicated a worse survival for patients with Ta stage and PDGFRa “high” expression (HR(95%CI) 2.35(1.11–5.00) Wald test p = 0.026) and T1 stage patients with ASMA “high” expression (HR(95%CI) 2.01(1.16–3.49) Wald test p = 0.013) (Fig. 2B). However, these finding did not remain significant after adjustment for multiple testing through Bonferroni correction (critical p-value for 15 performed tests p < 0.003). Of note, very similar results were obtained when looking at 5-year progression free survival in stage specified patient groups (Fig. 2B).

Taken together, none of the analyzed single stroma markers could be confirmed as independent prognostic marker within the UBC cohort when using a median-based cut-off. All tested stroma markers with exception of PDGFRa showed a strong correlation with tumor stage. Tumor stage and age group remained the strongest independent prognostic factors.

### Cluster analysis of stroma markers for refined patient stratification

**Figure 3:**
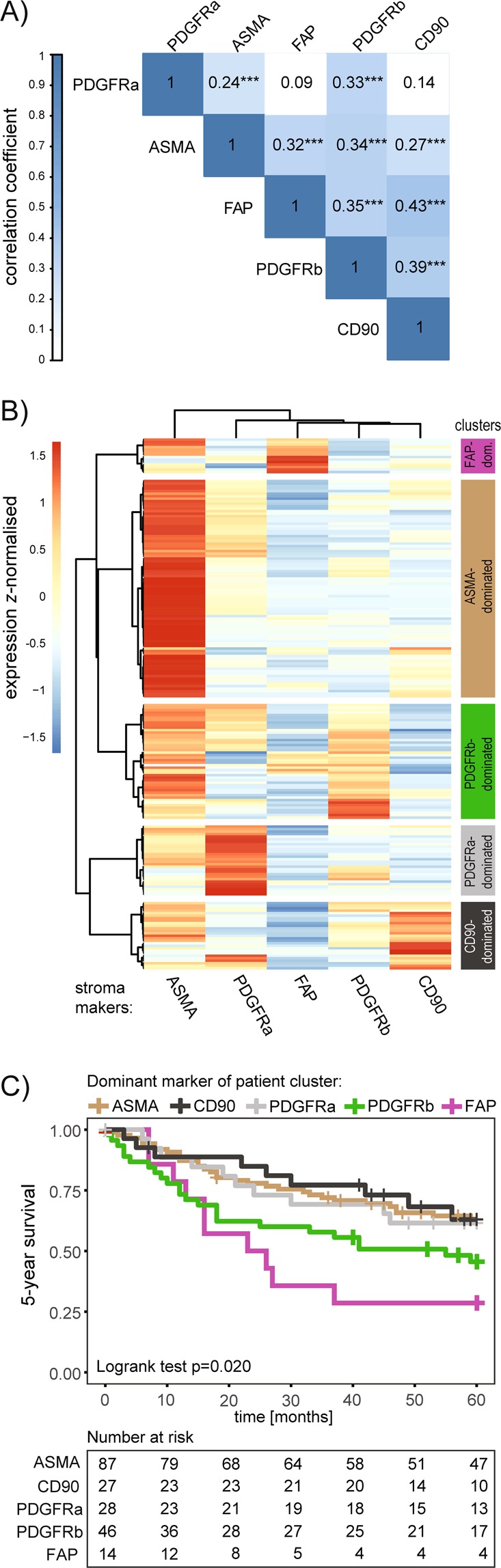
(**A**) Goodman and Kruskal’s gamma correlation analysis between the different stroma markers based on non-dichotomized scoring data. Numbers represent the *gamma* correlation coefficient. Only correlations with p < 0.01 are considered significant. Not significant observations are not color-coded. (**B**) Ward’s method based hierarchical clustering with Euclidean distance was performed on the non-dichotomized raw scoring data of the stroma markers. The heat-map represents the *z-*normalized expression levels of the markers. Each horizontal row represents an individual patient while each column is assigned to one of five markers. Five patient clusters are identified and named based on dominant expression of one of the analyzed stroma markers. (**C**) Kaplan–Meier analysis of the relationship between stroma-marker defined patient clusters and 5-year overall survival. P-value for overall curve difference is based on logrank test. The table indicates the number of individuals at risk at the given time points.

In order to investigate the relation of the stroma markers to each other, Goodman and Kruskal’s gamma correlation analysis was performed using the non-dichotomized raw scoring data (Fig. 3A). All stroma markers showed significant positive correlations between each other with the exception of PDGFRa and FAP as well as PDGFRa and CD90. Nevertheless, the correlation coefficients range from *gamma* = 0.43 (CD90 and FAP) to *gamma* = 0.24 (ASMA and PDGFRa) indicating rather weak correlations.

In the next step a hierarchical cluster analysis was performed aiming for an unsupervised approach to stratify and define patient groups taking into account all analyzed stroma markers simultaneously (Fig. 3B). Five patient clusters were identified. Some of the clusters were characterized by relatively clear dominant expression of one marker while others were not. However, for simplicity reasons we assigned the names to the cluster according to the most dominant marker inside the cluster. This terminology is used further in the text (Fig. 3B).

The identified clusters were subjected to survival analysis. Overall curve comparison indicated significant differences in 5-year overall survival between the clusters (logrank test p = 0.020) with the FAP- and PDGFRb-dominant patient cluster having the worst prognosis (Fig. 3C). Of note, the FAP-dominant cluster remained an independent significant prognostic marker in a multivariable Cox regression analysis (HR (95% CI) 2.25 (1.08–4.67), p = 0.030 Wald test) in comparison to ASMA as reference group) (Table 1). Interestingly, the multivariable analysis further revealed the CD90-dominant patient cluster as a good prognostic cluster, although not statistically significant (0.58 (0.27–1.25), p = 0.165 to ASMA as reference group). Similar results were obtained within a multivariable Cox regression analysis for 5-year progression free survival restricted to the non-muscle invasive patients (Ta and T1) (Supplementary Table 3).

**Table 1:** Multivariable analysis for 5-year survival data, including stroma-marker defined patient clusters.

n (included in regression model)	344 (202)	
	HR (95% CI)	p-value
**Age**		
≤72	1	**<0.001**
>72	3.47 (2.13–5.64)	
**Gender**		
female	1	0.753
male	1.09 (0.65–1.83)	
**Stage**		
Ta	1	
T1	1.72 (0.92–3.22)	0.092
T2 + 3 + 4	6.17 (3.04–12.52)	**<0.001**
**Grade**		
low (1–2 A)	1	0.362
high (2B-4)	1.48 (0.64–3.40)	
**Stroma-marker defined patient cluster, dominant marker:**		
ASMA	1	
CD90	0.58 (0.27–1.25)	0.165
FAP	2.25 (1.08–4.67)	**0.030**
PDGFRa	0.95 (0.46–1.98)	0.895
PDGFRb	1.33 (0.77–2.29)	0.308

Multivariable analysis using a Cox proportional hazards regression model including patient and clinical tumor characteristics as categorical variables as well as stroma-marker defined patient clusters. Hazard ratios (HR) for 5-year survival are presented with 95% confidence interval (CI) and p-values are based on Wald test.

Correlation analysis indicated a positive association between the CD90-, FAP- and PDGFRb-dominant clusters with tumor stage as well as with grade, which was not observed for the ASMA- and PDGFRa- dominant groups (Table 2).

**Table 2:** Associations between clinicopathological parameters and stroma marker defined patient clusters.

Dominant cluster marker	ASMA n (%)	CD90 n (%)	FAP n (%)	PDGFRa n (%)	PDGFRb n (%)	p-value*
**Age**						
≤72	46 (52.9)	14 (51.9)	5 (35.7)	17 (60.7)	20 (43.5)	0.488
>72	41 (47.1)	13 (48.1)	9 (64.3)	11 (39.3)	26 (56.5)	
**Gender**						
female	24 (27.6)	4 (14.8)	3 (21.4)	7 (25.0)	9 (19.6)	0.691
male	63 (72.4)	23 (85.2)	11 (78.6)	21 (75.0)	37 (80.4)	
**Stage**						
Ta	42 (48.3)	3 (11.1)	4 (28.6)	12 (42.9)	11 (23.9)	0.007
T1	33 (37.9)	19 (70.4)	7 (50.0)	10 (35.7)	22 (47.8)	
T2 + 3 + 4	12 (13.8)	5 (18.5)	3 (21.4)	6 (21.4)	13 (28.3)	
**Grade**						
low (1–2 A)	29 (33.3)	3 (11.1)	2 (14.3)	10 (35.7)	4 (8.7)	0.003
high (2B-4)	58 (66.7)	24 (88.9)	12 (85.7)	18 (64.3)	42 (91.3)	
**Recurrences**^**#**^						
no	12 (20.0)	3 (18.8)	1 (11.1)	8 (47.1)	5 (17.9)	0.281
few^$^	27 (45.0)	10 (62.5)	6 (66.7)	4 (23.5)	15 (53.6)	
frequent^†^	21 (35.0)	3 (18.8)	2 (22.2)	5 (29.4)	8 (28.6)	

Association between stroma-marker defined patient clusters and clinicopathological parameters analyzed by contingency tables.

^*^P-values are based on Fisher’s exact test or in case of tumor stage on Monte Carlo estimation.

^#^only non-muscle invasive Ta and T1 cases included (n = 130).

^$^<3 recurrences within 18 months; ^†^≥3 recurrences within 18 months.

In summary, this set of analyses demonstrates a refined stroma marker-based UBC patient stratification through cluster analysis and identifies a FAP-dominant patient cluster that acts as an independent marker for shorter 5-year survival.

### Associations between stroma-defined patient clusters and CD8a^positive^ T cell frequency

**Figure 4:**
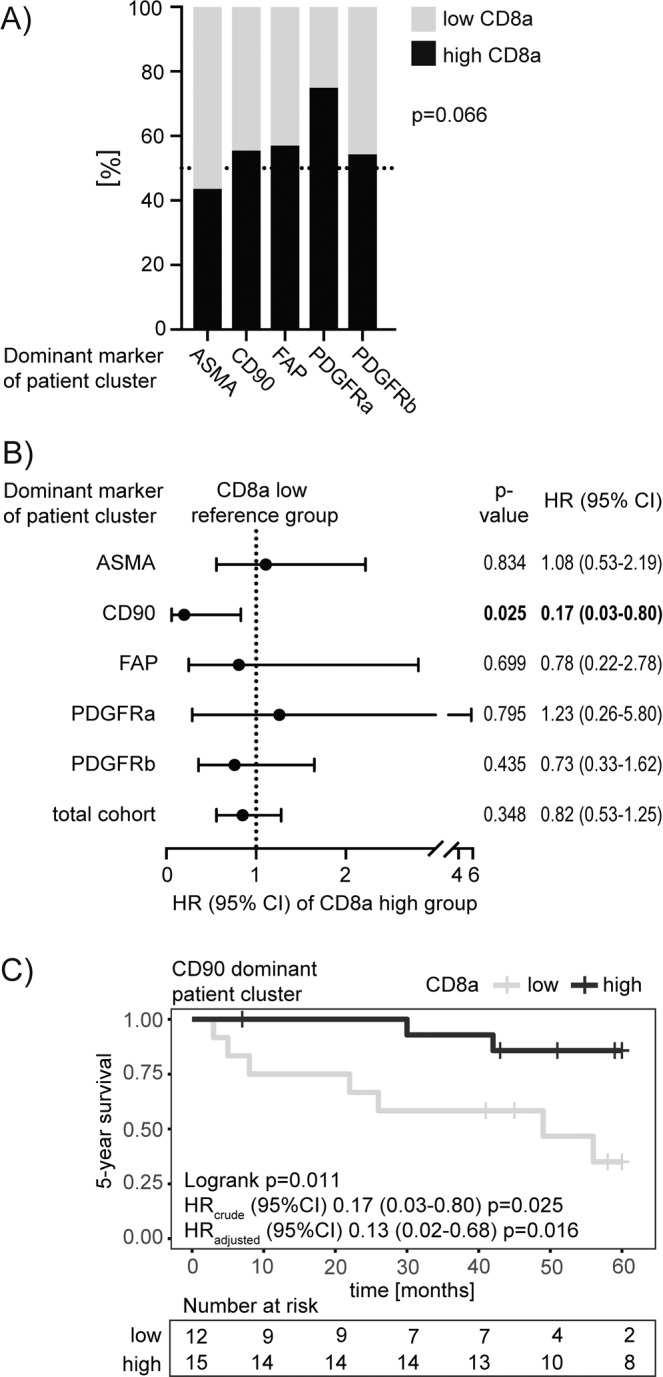
(**A**) Association between stroma-marker defined patient clusters and CD8a status based on congruency table analysis. P value is calculated by Fisher’s extact test. (**B**) Forest plot indicating overall survival hazard ratios (HR) with 95% confidence interval (CI) for patients with high numbers of CD8a in reference to those patients with low CD8a number within the whole population or stratified by the stroma-marker defined clusters. HR are based on cox proportional hazards regression models and Wald test (*p < 0.05 is highlighted in bold). (**C**) Kaplan–Meier analysis of the relationship between “low” (grey line) and “high” (black line) CD8a frequency and the 5-year overall survival within the CD90-dominant patient cluster. P-values from log-rank test are given to evaluate differences between the population survival curves. Table indicates the number of individuals at risk at the given time points. The unadjusted “crude” HR with 95% CI is based on Cox regression and Wald test. Adjusted HR included gender, age group, grade and stage as additional categorical variables in the multivariable (MV) regression model.

For better characterization of the different stroma-defined patient clusters, potential associations with tumor-infiltrating CD8a^positive^ T cells were investigated. Within the PDGFRa-dominant cluster a higher frequency of CD8a cells was observed, although not reaching statistical significance in congruency table analysis (p = 0.066 Fisher’s Exact test) (Fig. 4A).

While CD8a status alone had no prognostic significance within the whole cohort, overall survival analysis by cox regression models showed that within the CD90-dominant patient cluster a high frequency of CD8a^positive^ T cells was associated with a longer 5-year survival rate (logrank p = 0.011; HR (95% CI) 0.17 (0.03–0.80), Wald test p = 0.025) (Fig. 4B,C). This association remained significant in a multivariable analysis including age group, gender, stage and grade (Fig. 4B,C). Five-year progression free survival analysis, restricted to the non-muscle invasive patients, indicated a very similar tendency, although not significant (Supplementary Fig. 4). No association between the frequency of CD8a cells and survival was observed in the other stroma-defined patient clusters. Of note, similar analyses for the prognostic potential of CD8a within the single stroma marker defined “low” and “high” patient groups did not show significant effects (data not shown).

Together, these correlative analyses point towards the existence of different stroma-cell subgroups in UBC, where CD8a status shows differential prognostic significance.

## Discussion

The past years, stromal cells have been recognized as not only passive bystanders, but active key players in tumor progression^50^. While experimental functional studies have described tumor-supportive as well as tumor -restraining functions of CAFs in the UBC context, clinical evidence for the existence of different functional subsets remains sparse^5,15–17,51^. The presented study has analyzed the interaction of the five stromal markers ASMA, CD90, FAP, PDGFRa and –b with survival and histopathological data within a UBC cohort. While all of the markers, with exception of ASMA, were associated with adverse prognosis in univariable analysis, none of the investigated markers remained a statistically significant independent prognostic marker in a multivariable Cox regression model. This is likely to be explained by the strong positive correlation of the stroma markers with tumor stage, indicating that higher abundance and expression intensity of the stromal markers occurs with muscular invasion.

Aiming for a more comprehensive analytic approach, which allows a refined cut-off definition for each stroma marker, cluster analysis and subsequent multi-marker-based survival analysis was performed. Through the hierarchical cluster analysis, expression ratios between the different stroma markers are taken into account and thereby patient subgroups can be stratified based on information of all stroma metrics. Indeed, a FAP-dominant patient cluster was identified, which remained an independent bad prognosis marker in a multivariable model. This data gives evidence for how multi-marker-based analyses can refine single marker analyses.

The conclusion is supported by a very recent study also on UBC, which as well identified a multi-marker-based expression signature composed of FAP and the basal markers CK5/6 and CD44^52^. This multi-marker panel outperformed single marker analyses as a strong independent prognostic factor for disease-specific survival and nodal invasion^52^. From a biological point of view, the finding that FAP-dominant patient cluster represents the worst prognostic patient group, is in line with previous data from experimental as well as correlative studies in other solid tumors. FAP is described as a bad prognostic marker in colorectal cancer^53,54^, ovarian cancer^55^ and pancreatic ductal adenocarcinoma^56^ and experimental studies have linked FAP positive fibroblasts to immunosuppressive functions^28–31^. However, FAP-positive fibroblasts as bad prognostic marker in UBC are just being recognized^52^.

Notably, PDGFRa expression did not show an association with tumor stage or grade, neither in the single-marker analysis nor for the PDGFRa-dominant patient cluster. Three recent studies on breast cancer, suggest that PDGFRa expressing fibroblasts are resident fibroblasts also present in normal tissue^33–35^, and that these fibroblasts are linked to a good prognosis^33,34^.

Of comment, the presented analyses are based on single immunohistochemistry stainings and therefore conclusion on potential marker co-expression in fibroblasts cannot be drawn. It would be interesting to investigate the degree of marker co-expression in fibroblasts as well as the co-existence of different marker-defined fibroblast subsets using the novel multiplexing immunofluorescence-based techniques. As these techniques enable analysis down to the single cell level and therewith allow stromal-cell subtyping *in situ*, they could give the possibility of even more refined patient stratification. Nevertheless, such studies have not yet been performed.

In context of the increasing recognition of stroma cell mediated immune modulation, this study has also investigated associations of the stroma-marker defined patient clusters with tumoral CD8a-positive cells. Although a clear significant interaction could not be demonstrated, it was interesting observing that a high infiltration of CD8a cells was only associated with a better prognosis within the CD90-dominant patient cluster. The role of CD90-positive fibroblasts in cancer progression and especially immune cell regulation is still mostly unknown and occasional reports are contradicting^57^ especially with regard to their potential antigen presenting functions^46–48^. However, the given observations from this study support the hypothesis of functional differences between fibroblast subsets and experimental studies addressing this point would therefore be of high interest.

Limitations of the current study are the use of a retrospective patient TMA and therefore validation of the indicated findings on independent UBC cohorts are highly motivated. Especially conclusion on the survival associations for CD8a positive lymphocytes within the cluster-defined patient subgroups should be further validated given that some of the patient subgroups are relatively small. Data on the intravesical treatment of the presented patient cohort is incomplete and relatively heterogeneous among the reported cases, including treatment with Bacillus Calmette–Guérin (*BCG*) immunotherapy, which might further limit conclusions on the immunomodulatory functions of fibroblast subsets.

Altogether, the presented data provides a comprehensive analysis of the association of multiple stroma markers with the 5-year overall survival of UBC patients. A FAP-dominant patient cluster is identified as a significant independent prognostic marker. Notably, correlative analyses with histopathological tumor characteristics including the infiltration of CD8a-positive cells point toward functional distinct marker-defined fibroblast subsets in UBC.

## Materials and Methods

### Patient cohort

The 357 Uppsala University Hospital patients included in this study were diagnosed with bladder cancer between 1984 and 2005. The tumor material comprises a wide-range tissue microarray (TMA) of prospectively collected primary tumors with clinical data being available for 344 UBC patients (115 Ta tumors, 116 T1 tumors and 113 T2-T4) (Fig. 1) and a median follow-up time of 44.5 months. Five-year overall survival was used as main endpoint and was calculated from the date of surgery to date of death or last follow-up. At follow up of the prospective material the non-muscle invasive patients were categorized as having none, few or frequent recurrences. Definition of few recurrences was less than three recurrent tumors within 18 months, whereas frequent recurrences were three or more recurrences within the same time period. For the TMA-production, representative tissue areas were identified by a pathologist classifying the tumors according to the WHO grading system of 2004^58^.

The TMA was constructed using an automated instrument, ATA-27 (Beecher Instruments, WI, USA). All tissue samples were represented in duplicate tissue cores (1 mm). The cohort and TMA has been published previously^11,59–62^.

### Ethics approval and consent to participate

Protein profiling of samples from the described patient cohort was approved by the regional ethical review board of Uppsala (reference number 2015/143). The same ethical review board waived the need for written informed consent for this retrospective study. The study was performed in accordance with the Declaration of Helsinki.

### Immunohistochemistry (IHC)

IHC was performed on 4 µm sections of the TMA blocks. PDGFRa, PDGFRb and CD90 staining was done on the Ventana Benchmark Discovery NexES V10.6 autostainer system (Ventana Medical Systems, Oro Valley, Arizona, United States). The rabbit monoclonal anti-PDGFRb antibody (clone 28E1, #3169 Cell Signaling, Danvers MA, US; 1:100 dilution), the rabbit monoclonal anti-PDGFRa antibody (clone D13C6, #5241 Cell Signaling, Danvers MA, US; 1:100 dilution) and the monoclonal rabbit anti-CD90 antibody (clone EPR3132, #ab92574, Abcam, Cambridge, UK) were diluted in Antibody Diluent Buffer (Antibody diluent, Ventana, Tuscon, Arizona, US). An extended antigen retrieval step with Cell Conditioning Buffer 1 (CC1) (Ventana) was included for the PDGFRa and CD90 staining and with pH10 Tris buffer (Sigma-Aldrich and Merck Kgaa, Darmstadt, Germany) for the PDGFRb staining. The staining protocol was performed with the Discovery OmniMap anti-rabbit HRP (RUO) detection kit (Ventana), including incubation with the primary antibody for 1 h and incubation with the secondary anti-rabbit HRP antibody for 32 min. Counterstaining was done with Hematoxylin II for 10 min and subsequent bluing for 6 min (Ventana). After dehydration, sections were mounted with Pertex® Mounting Medium (HistoLab, Askim, Sweden).

The FAP/CD8a doublestaining included the FAP staining on the Ventana Benchmark Discovery autostainer system using the rat anti-FAP antibody (clone D8; #MABS1001, Vitatex, Stony Brook, NY) diluted 1:150 in Background Reducing Antibody Diluent (DAKO, Glostrup, Danmark). An extended antigen retrieval step with Cell Conditioning Buffer 1 (CC1) (Ventana) was included and staining was performed using the UltraMap anti-rat HRP (RUO) detection kit (Ventana) including incubation with the primary antibody for 1 h and with the secondary anti-rat HRP antibody for 16 min. No counterstaining was performed. Slides were removed from the Ventana machine and washed 5 min in PBS-Tween20 (phosphate buffered saline plus 0.05% Tween20) and blocked for 20 minutes with Dako Protein block Serum-free (DAKO). Mouse monoclonal anti-CD8a antibody (clone C8/144B, #M7103, Dako) was diluted 1:100 in Dako Real Antibody Diluent (Dako) and applied onto the slides for over-night incubation at 4 °C. After washing, the sections were incubated with alkaline phosphatase conjugated secondary anti-mouse antibody (ImmPRESS™-AP Polymer Anti-Mouse IgG, MP-5402, Vector Laboratories, Burlingame, CA) for 30 min at room temperature and developed with Vector® Blue AP Substrate Kit (SK-5300, Vector Laboratories). Sections were mounted using Faramount Aqueous Mounting Medium (DAKO).

The ASMA staining was performed manually. Sections were deparaffinized in Histo-Clear (National Diagnostics, Atlanta, GA, USA) 3 × 5 minutes and washed and rehydrated in a serial dilution of ethanol (100, 95 and 70%) for 2 × 5 minutes each, and finally submerged in dH_2_O. Antigen retrieval was performed at pH9 (Target retrieval solution, DAKO) in a decloaking chamber (Biocare Medical, Walnut Creek, CA, USA) at 110 °C for 5 minutes. Samples were blocked for 20 minutes with Dako Protein block Serum-free (DAKO), before primary antibody incubation with a 1:100 dilution of anti-ASMA (clone 1A4, M0851, DAKO) for 1 hour at room temperature. Slides were washed in PBS-Tween20 and incubated with horseradish-peroxidase conjugated secondary anti-mouse antibody (ImmPRESS™-HRP Polymer Anti-Mouse IgG MP-7402, Vector Laboratories) for 1 hour at room temperature. Slides were washed with PBS-Tween20 before development with DAB Peroxidase Substrate Kit (SK-4100, Vector Laboratories). Slides were dehydrated in a serial dilution of ethanol (70, 95, 100%). Mounting was done with VectraMount Permanent Mounting Medium (Vector Laboratories).

The PDGFRa and –b antibodies have been validated and described previously and cross-detection of the two related receptors has been excluded^33,36^. The ASMA^39,63–65^, CD90^66,67^, FAP^54,68^ and CD8a^65,69,70^ antibodies are widely used in IHC by several groups and have been published previously. Cross-reactivity of these antibodies has been validated by Western Blot analysis by the manufacturers and all antibodies are recommended for IHC according to the manufacturer’s datasheet.

### Marker evaluation

Stained slides were scanned on the Hamamatsu Nanozoomer S60 (Hamamatsu, Hamamatsu City, Japan). Two independent raters (CS and AM), blinded to the clinical data, scored the cores for average intensity as well as the positive stroma fraction following a four-grade scale from negative/none (0+), weak/low (1+), moderate (2+) to strong/high (3+). In case of score discordance between the duplicate tissue cores per patient on the TMA the higher score was assigned. For all stroma markers, the product of average intensity and positive stroma fraction was calculated per rater in order to obtain the overall score. The frequency of CD8 positive cells was assessed on a five-grade scale from none (0), scattered (1), few (2), moderate (3) and high (4) in the tumor and stroma compartment separately. The overall CD8 score per rater was calculated as the sum of the epithelial and stromal CD8a scores was formed per rater.

To assess the degree that raters provided consistency in their scorings the inter-rater agreement was calculated with Cohen’s kappa (squared weightage)^49^ using the *irr* package for R. For subsequent analyses, the average score between the two raters was calculated resulting in a data range from 0 to 9 with 0.5 intervals (see also Supplementary Figs. 1 and 2). For Kaplan Meier and Cox regression analyses on single marker data the scoring data was split at the median in order to obtain two patient groups, as equally sized as possible, referred to as “low” and “high” group (Supplementary Figs. 1 and 2). For tumor stage specific analyses data dichotomization was performed within each stage group.

### Statistics

Statistical analyses were carried out using SPSS 22 (SPSS Inc., Chicago, IL) and R Studio software (version 1.0.143). Five-year overall survival and 5-year progression free survival were used as endpoints and were calculated from the date of surgery to date of death or last follow-up or date of surgery to date of recurrence, death or last follow-up, respectively. Kaplan-Meier analysis with logrank test was applied to compare survival between different patient groups using the *survminer* package for R. Hazard ratios (HR) were calculated in SPSS using Cox proportional hazards regression models and Wald test. Multivariable Cox regression models including gender, age, stage and grade as categorical covariates were performed to calculate adjusted HR of the corresponding stroma markers. For Cox regression analysis the assumption of proportional hazard was verified graphically through evaluation of parallelism of the log(−log(S(t))) versus time plot as well as statistically through the Schoenfeld Residuals Test.

Associations between stromal marker status and clinico-pathological parameters or CD8a data were analyzed by contingency tables with Fishers’ exact test or in case of “Stage” with the Mantel-Haenszel linear-by-linear association χ^2^-test. Goodman and Kruskal’s *gamma* correlation analysis between the different stroma markers was performed on the non-dichotomized raw scores with pairwise deletion in case of missing data. All correlation analyses were performed in SPSS. Ward’s (Ward.D2) hierarchical clustering method with Euclidean distance was done with the *pheatmap* package in R.

All given 95% confidence intervals (CI) and statistical tests were two-sided. P values < 0.05 were considered significant if not indicated otherwise. In case of multiple testing, Bonferroni correction was applied to adjust the critical p-value by dividing 0.05 through the number of performed tests.

## Supplementary information

Supplementary material

## Data Availability

The anonymized clinical data table including the raw and dichotomized histological scores is available from the corresponding author on request.
