# Supplementary material for: Fibroblasts in urothelial bladder cancer define stroma phenotypes that are associated with clinical outcome
Source: Sci Rep. 2020 Jan 14;10:281. doi: 10.1038/s41598-019-55013-0 (PMC6959241; doi:10.1038/s41598-019-55013-0)
Supplement: Supplementary file 1 — Supplementary material [file 41598_2019_55013_MOESM1_ESM.pdf]

Supplementary Material

“Fibroblasts in urothelial bladder cancer define stroma phenotypes that are associated with clinical outcome”

Artur Mezheyeuski, Ulrika Segersten, Lina Wik Leiss, Per-Uno Malmström, Jiri Hatina, Arne Östman, Carina Strell

**Supplementary table 1: Multivariable analysis for 5-year survival data, including stromal marker expression**										
n (included in regression model)	344 (341)		344 (333)		344 (343)		344 (263)		344 (247)	
	HR (95% CI)	p-value	HR (95% CI)	p-value	HR (95% CI)	p-value	HR (95% CI)	p-value	HR (95% CI)	p-value
**Age**										
≤ 72	1	**<0.001**	1	**<0.001**	1	**<0.001**	1	**<0.001**	1	**<0.001**
> 72	3.05 (2.19-4.27)		3.23 (2.32-4.61)		3.08 (2.21-4.29)		3.37 (2.22-5.12)		3.18 (2.13-4.76)	
**Gender**										
female	1	0.263	1	0.387	1	0.336	1	0.758	1	0.926
male	1.23 (0.85-1.78)		1.18 (0.82-1.70)		1.20 (0.83-1.72)		1.07 (0.69-1.67)		1.02 (0.66-1.58)	
**Stage**										
Ta	1	**<0.001**	1	**<0.001**	1	**<0.001**	1	**<0.001**	1	**<0.001**
T1	1.49 (0.91-2.45)		1.58 (0.95-2.63)		1.48 (0.90-2.45)		1.48 (0.87-2.50)		1.68 (0.93-3.06)	
T2+3+4	4.51 (2.73-7.46)		5.31 (3.10-9.09)		4.53 (2.62-7.84)		4.56 (2.54-8.16)		4.86 (2.61-9.05)	
**Grade**										
low (1-2A)	1	0.428	1	0.295	1	0.441	1	0.338	1	0.638
high (2B-4)	1.27 (0.71-2.27)		1.39 (0.75-2.56)		1.26 (0.70-2.26)		1.36 (0.73-2.53)		1.19 (0.57-2.51)	
**ASMA**[#]										
low	1	0.531	-	-	-	-	-	-	-	-
high	1.11 (0.81-1.52)		-	-	-	-	-	-	-	-
**CD90**[§]										
low	-	-	1	0.138	-	-	-	-	-	-
high	-	-	0.77 (0.54-1.09)		-		-		-	
**FAP**[¶]										
low	-	-	-	-	1	0.969	-	-	-	-
high	-	-	-	-	1.01 (0.70-1.46)		-		-	
**PDGFRa**[‡]										
low	-	-	-	-	-	-	1	0.278	-	-
high	-	-	-	-	-	-	1.24 (0.84-1.83)		-	-
**PDGFRb**[$]										
low	-	-	-	-	-	-	-	-	1	0.144
high	-	-	-	-	-	-	-	-	1.33 (0.91-1.93)	

Supplementary table 1: Multivariable analysis using a cox proportional hazards regression model including patient and clinical tumor characteristics as well as single stroma markers dichotomized through median cut-off as categorical variables. Hazard ratios (HR) for 5-year survival are presented with 95% confidence interval (CI) and p-values are based on Wald test.

data missing for 3 patients; § data missing for 11 patients; ¶ data missing for 1 patient; ‡ data missing for 81 patients; $ data missing for 97 patients

**Supplementary table 2:** Clinicopathological parameters within stroma marker defined patient group.															
	**ASMA**[#]			**CD90**[§]			**FAP**[¶]			**PDGFRa**[‡]			**PDGFRb**[$]		
	low	high	p*	low	high	p*	low	high	p*	low	high	p*	low	high	p*
	**n (%)**	**n (%)**		**n (%)**	**n (%)**		**n (%)**	**n (%)**		**n (%)**	**n (%)**		**n (%)**	**n (%)**	
**Age**															
≤ 72	80 (46.5)	85 (50.3)	0.516	88 (54.0)	71 (41.8)	**0.028**	94 (52.2)	73 (44.8)	0.194	65 (52.4)	65 (46.8)	0.388	70 (53.4)	55 (47.4)	0.374
> 72	92 (53.5)	84 (49.7)		75 (46.0)	99 (58.2)		86 (47.8)	90 (55.2)		59 (47.6)	74 (53.2)		61 (46.6)	61 (52.6)	
**Gender**															
female	37(21.5)	46 (27.2)	0.256	33 (21.5)	45 (26.5)	0.307	36 (20.0)	47 (28.8)	0.060	31 (25.0)	30 (21.6)	0.560	37 (28.2)	21 (18.1)	0.071
male	135 (78.5)	123 (72.8)		128 (78.5)	125 (73.5)		144 (80.0)	116 (71.2)		93 (75.0)	109 (78.4)		94 (71.8)	95 (81.9)	
**Stage**															
Ta	62 (36.0)	51 (30.2)	**0.001**	76 (46.6)	31 (18.2)	**<0.001**	92 (51.1)	22 (13.5)	**<0.001**	50 (40.3)	56 (40.3)	**0.141**	51 (38.9)	26 (22.4)	**0.001**
T1	74 (43.0)	42 (24.9)		52 (31.9)	61 (35.9)		73 (40.6)	43 (26.4)		58 (46.8)	46 (33.1)		52 (39.7)	46 (39.7)	
T2+3+4	36 (20.9)	76 (45.0)		35 (21.5)	78 (45.9)		15 (8.3)	98 (60.1)		16 (12.9)	37 (26.6)		28 (21.4)	44 (37.9)	
**Grade**															
low (1-2A)	40 (23.3)	40 (23.7)	1.000	52 (31.9)	22 (12.9)	**<0.001**	65 (36.1)	16 (9.8)	**<0.001**	34 (27.4)	41 (29.5)	0.785	40 (30.5)	14 (12.1)	**0.001**
high (2B-4)	132 (76.7)	129 (76.3)		111 (68.1)	148 (87.1)		115 (63.9)	147 (90.2)		90 (72.6)	98 (70.5)		91 (69.5)	102 (87.9)	
**Recurrence**∫															
no	25 (22.1)	13 (18.3)	0.677	22 (21.4)	14 (19.5)	0.915	31 (24.0)	8 (14.3)	0.826	13 (15.1)	24 (28.9)	0.091	20 (23.6)	13 (22.8)	0.642
few ¶	51 (45.2)	34 (47.9)		48 (46.6)	35 (48.6)		53 (41.1)	32 (57.1)		43 (50.0)	35 (42.2)		37 (43.5)	29 (50.9)	
frequent "	37 (32.7)	24 (33.8)		33 (32.0)	23 (31.9)		45 (34.9)	16 (28.6)		30 (34.9)	24 (28.9)		28 (32.9)	15 (26.3)	

Supplementary table 2: Association between single stroma markers dichotomized through median cut-off and clinicopathological parameters analyzed by contingency tables.

*P-values are based on Fisher's exact test or in case of tumor stage and recurrence on the Mantel-Haenszel linear-by-linear association χ^2-test.

data missing for 3 patients; § data missing for 11 patients; ¶ data missing for 1 patient; ‡ data missing for 81 patients;

$ data missing for 97 patients

∫ restricted to non-muscle invasive cases with Ta and T1 (data available for 185 patients, data missing for 46 patients)

¶ <3 recurrences within 18 months

" ≥3 recurrences within 18 months

Supplementary Table 3: Multivariable analysis for 5-year progression free survival, including stroma-marker defined clusters for patients with non-muscle invasive disease		
n included in regression model	163	
	HR (95% CI)	p-value
**Age**		
≤ 72	1	
> 72	1.94 (1.12 – 3.36)	**0.018**
**Gender**		
female	1	
male	1.25 (0.63 – 2.50)	0.520
**Stage**		
Ta	1	
T1	1.62 (0.85 - 3.09)	0.147
**Grade**		
low (1-2A)	1	
high (2B-4)	1.51 (0.66 - 3.47)	0.330
**Stroma-marker defined patient cluster, dominant marker:**		
ASMA	1	
CD90	0.98 (0.42 – 2.28)	0.960
FAP	2.52 (0.71 – 2.70)	**0.035**
PDGFRa	1.09 (0.44 – 2.71)	0.860
PDGFRb	1.38 (0.71 - 2.70)	0.343

Supplementary table 3: Multivariable analysis using a cox proportional hazards regression model including clinical tumor characteristics as categorical variables as well as stroma-marker defined patient clusters for patients with non-muscle invasive disease (Ta and T1). Hazard ratios (HR) for 5-year progression free survival are presented with 95% confidence interval (CI) and p-values are based on Wald test.

Supplementary Figure 1

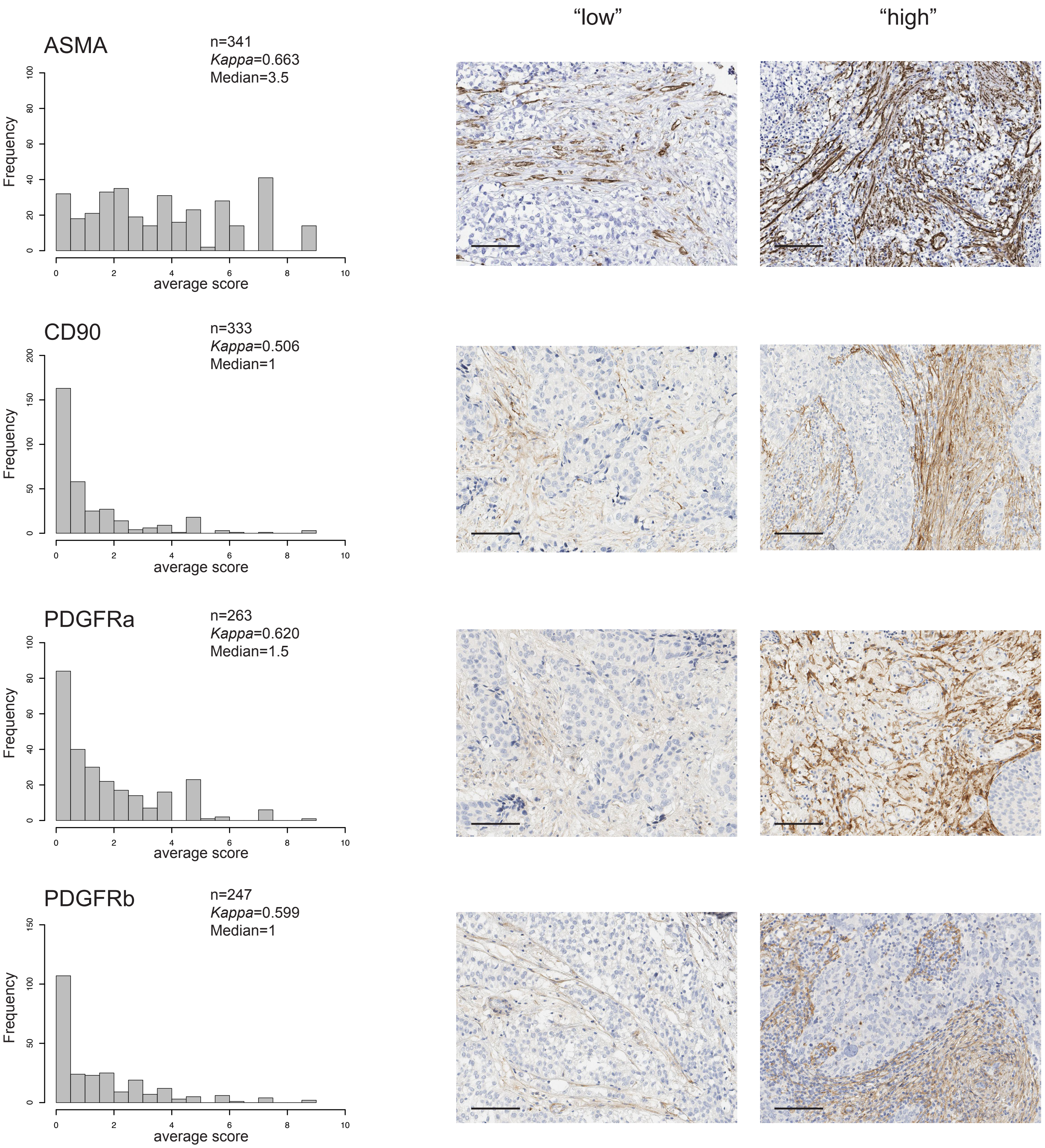

Supplementary Figure 1

Histogram of the overall score distribution for ASMA, CD90, PDGFRa and PDGFRb after averaging of the two raters (left panel).

Cohen’s *kappa* coefficient is given as measurement for inter-rater agreement.

The right panel shows representative immunohistochemical stainings of the corresponding stroma marker.

Scale bar represents 100 um.

Supplementary Figure 2

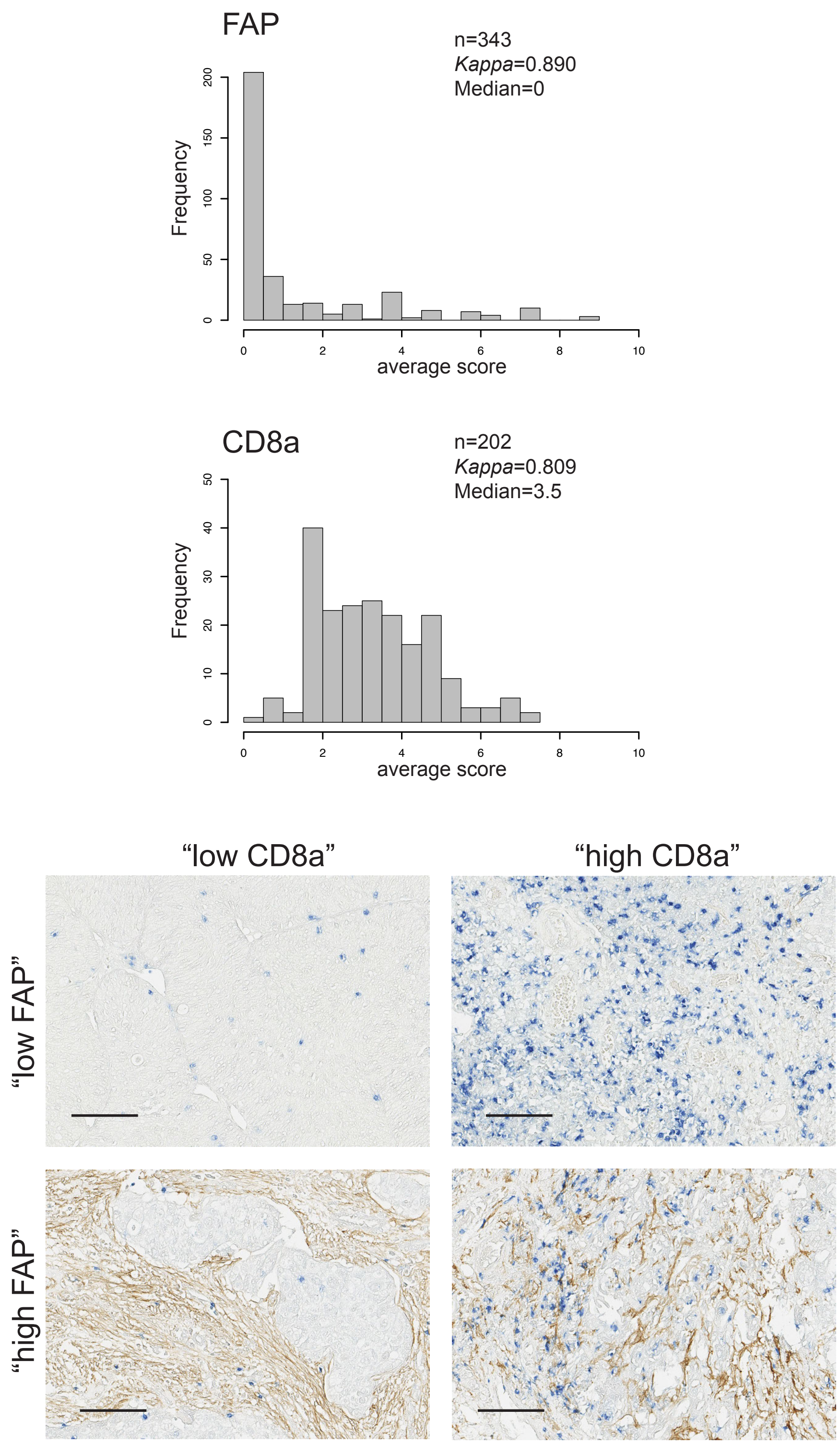

Supplementary Figure 2

Histogram of the overall score distribution for FAP and CD8 after averaging of the two raters (upper two panels).

The lower images shows representative immunohistochemical doublestainings of FAP (stained brown with DAB) and CD8a (stained in blue with Vector Blue). No counterstain was applied.

Cohen’s *kappa* coefficient is given as measurement for inter-rater agreement.

Scale bar represents 100 um.

For CD8a the histogram represents only those 202 cases and their cut-off that were included in the study (those cases where also data was complete for all five stroma markers) .

Supplementary Figure 3

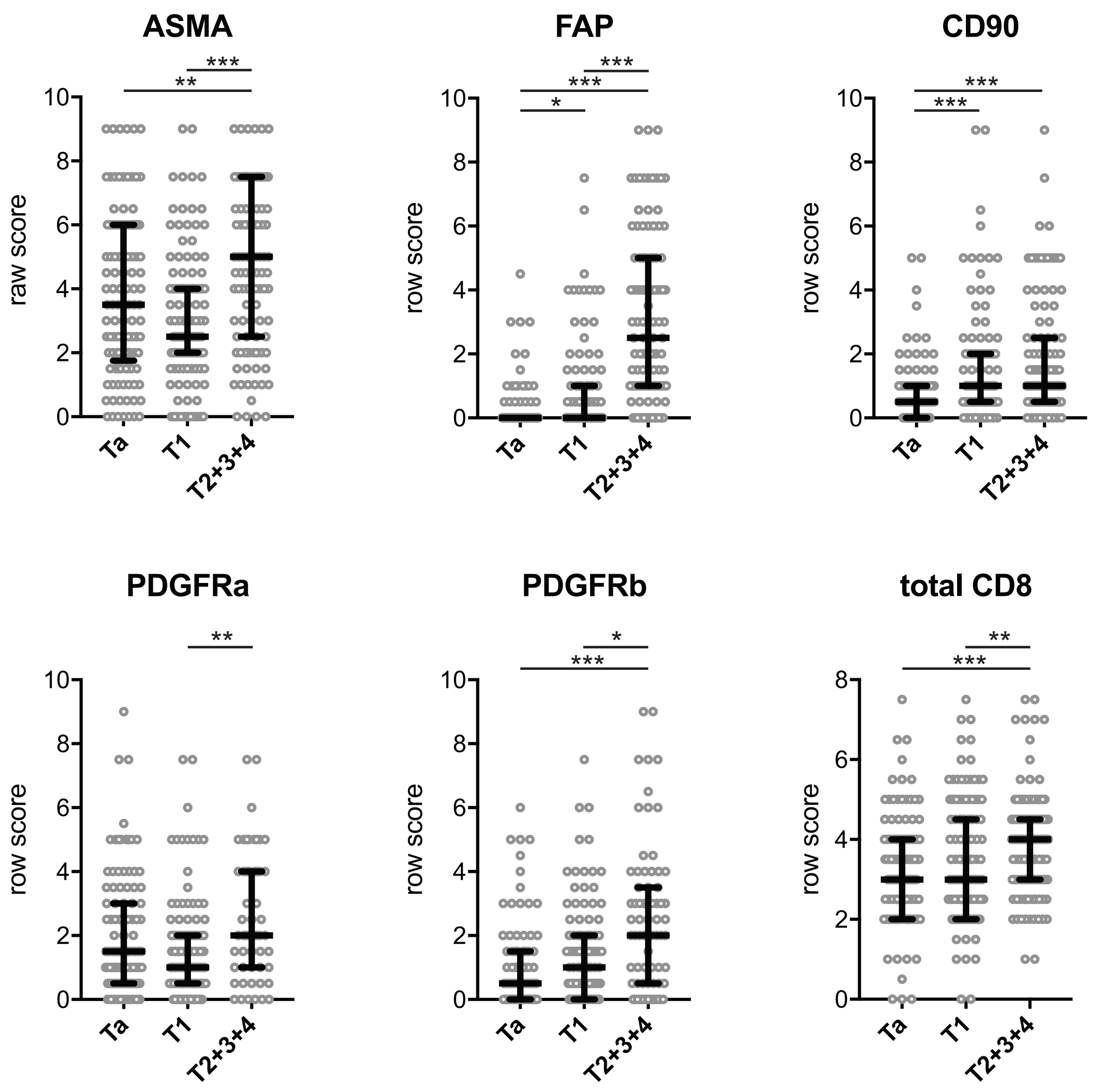

Supplementary Figure 3
Comparisons of raw score distributions between the separate tumor stage groups. P-values from group comparisons are based on Kruskal-Wallis test with Dunn’s correction for multiple tests. * p<0.05; ** p<0.01; ***p<0.001

Supplementary Figure 4

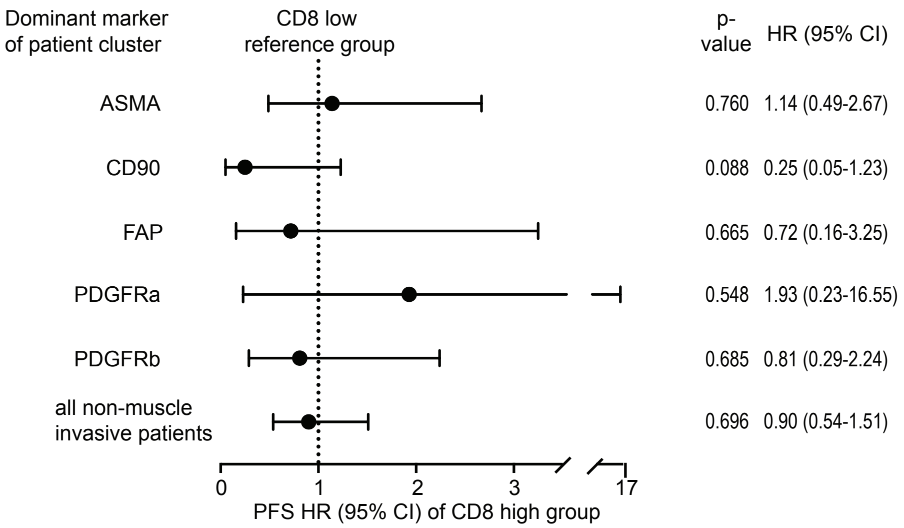

Supplementary Figure 4
Forest plot indicating 5-year progression free survival (PFS) hazard ratios (HR) with 95% confidence interval (CI) for patients with high numbers of CD8 in reference to those patients with low CD8 number within the whole population or stratified by the stroma-marker defined clusters. HR are based on cox proportional hazards regression models and Wald test.
